# The Motivations for Consumption of Edible Insects: A Systematic Review

**DOI:** 10.3390/foods11223643

**Published:** 2022-11-15

**Authors:** Sofia G. Florença, Raquel P. F. Guiné, Fernando J. A. Gonçalves, Maria João Barroca, Manuela Ferreira, Cristina A. Costa, Paula M. R. Correia, Ana P. Cardoso, Sofia Campos, Ofélia Anjos, Luís Miguel Cunha

**Affiliations:** 1 Faculty of Food and Nutrition Sciences, University of Porto, 4200-465 Porto, Portugal; 2 CERNAS Research Centre, Polytechnic Institute of Viseu, 3504-510 Viseu, Portugal; 3 Molecular Physical-Chemistry R&D Unit, Department of Chemistry, University of Coimbra, 3004-535 Coimbra, Portugal; 4 Polytechnic of Coimbra, Coimbra Agriculture School, Bencanta, 3045-601 Coimbra, Portugal; 5 Health Sciences Research Unit: Nursing (UICISA: E), Polytechnic Institute of Viseu, 3504-510 Viseu, Portugal; 6 CIDEI-IPV Research Centre, Polytechnic Institute of Viseu, 3504-510 Viseu, Portugal; 7 School of Agriculture, Polytechnic Institute of Castelo Branco, 6001-909 Castelo Branco, Portugal; 8 Forest Research Centre, School of Agriculture, University of Lisbon, 1349-017 Lisbon, Portugal; 9 GreenUPorto–Sustainable Agrifood Production Research Centre/INOV4Agro, Departamento de Geociências, Ambiente e Ordenamento do Território (DGAOT), Faculty of Sciences, University of Porto, 4485-646 Vila do Conde, Portugal

**Keywords:** edible insects, determinants, consumer, perception, acceptance, Western countries, insect-eating countries, PRISMA

## Abstract

The consumption of edible insects (EI) is traditional in many parts of the world, but not in others. In fact, despite globalization and the multiple advantages pointed out about the consumption of EI, there are still many countries where entomophagy is seen with disgust and aversion. This systematic review aimed to examine the motivations that influence the consumption of EI in diverse cultures and understand if there are differences between Western countries (WC) and insect-eating countries (IEC). It further evaluated whether the degree of acceptability was influenced by the form of consumption of the insects (eating whole insects or foods containing insects). This literature review was conducted in November 2021 within three databases, Web of Science, PubMed and Scopus, according to the Preferred Reporting of Items for Systematic Reviews and Meta-analysis and using PRISMA directives. From a total of 245 studies, 31 were selected to be included in this review, based on the inclusion criteria defined: only original research articles, from 2010 or beyond, and written in English. The results indicated that the main motivations that determine the consumption of EI are related to gender, age, sustainability, nutritional value, sensory attributes, tradition/culture, food neophobia, disgust and familiarity/past experiences. Moreover, whereas in IEC, there is a greater focus on factors related to sensory attributes, availability, affordability and preferences, in WC, there is a bigger emphasis on determinants such as nutritional value, sustainability, benefits, familiarity/past experience, tradition/culture, food neophobia and disgust. Finally, it was observed that people in WC are more willing to accept food products containing insects rather than the whole insect, which is one of the most promising points to be addressed in the future. Overall, this review highlights that there are numerous factors influencing the consumption of edible insects, and differences between WC and IEC are clear in what concerns the motivations of consumers. Hence, targeting market segments and consumers’ characteristics has to be present when designing strategies to incentivize the consumption of EI in WC as a part of a global strategy for sustainability of food systems.

## 1. Introduction

It is predicted that by 2050, as a result of the exponential increase in the global population, the world will require 70% more food, creating a heavy pressure on the limited natural resources and highlighting pre-existing problems regarding the loss of biodiversity, emission of greenhouse gases (GHG), and deforestation [1,2,3]. 

Food is fundamental for the survival of humans; however, the current food system utilizes over 30% of land, 70% of drinking water and 20% of energy, deteriorating natural resources and ecosystems. To produce enough food for the future generations and, at the same time, maintain a healthy environment, it is necessary to establish food systems that are sustainable. A sustainable food system is defined as being a structure that “ensures food security and nutrition for all in such a way that the economic, social and environmental bases to generate food security and nutrition of future generations are not compromised” [1,2,3,4,5]. 

The new demands for food and animal protein contribute extensively to climate change and environmental degradation. These effects can be minimized by shifting to healthier and more sustainable diets. More recently, a new solution of a sustainable source of protein has been gaining attention: edible insects (EI) [2,6]. 

The practice of consuming insects, entomophagy, has been a part of the culture and tradition of approximately two billion people in countries of Africa, Asia, Latin America and Oceania. There are more than 1900 insect species documented as edible, being a source of nutrition, health, environmental sustainability and livelihood [3,7]. 

One of the important aspects of EI is their nutritional and health value. Insects’ nutritional composition depends on numerous factors such as insect species, gender, stage of development, type of feed and processing method, among others. The work by Meyer-Rochow et al. [8] compares the nutritional composition of different species of insects and also that of different species belonging to the same genus, and refers that one aspect that can also influence the composition is the rearing system, including the feed and ecological situation. They are a source of energy, protein, amino acids, essential fatty acids, fiber, minerals, such as potassium, calcium, iron, magnesium, selenium, zinc, copper and phosphorus, and vitamins such as riboflavin, pantothenic acid and biotin [3,9,10,11]. Insects’ nutrient profile, as well as the presence of bioactive peptides with antioxidant and antimicrobial properties, may bring some additional health benefits by potentially improving gastrointestinal health, increasing immunity, decreasing the risk of bacterial infections and even preventing and managing chronic diseases such as cancer and cardiovascular diseases by reducing chronic inflammation [12]. 

For environmental sustainability, it is crucial that organisms consume fewer resources per amount of animal protein. In this regard, EI require far less feed when compared with chicken, pork or beef to produce 1 kg of weight, therefore being efficient convertors of feed to food. In general, the feed-to-meat conversion for crickets is twice as efficient as chicken, four times more efficient than pigs and twelve times more than beef [3,6,7]. One other benefit of insects is that some species bioconvert organic waste with high efficiency, which can greatly reduce organic pollution, highlighting the ability to breed insects on organic side streams. Farming insects has other advantages such as lower land use and minimal water usage. It was estimated that about 1 hectare of land used to yield an amount of protein from mealworms corresponds to 2–3.5 hectares for the same quantity of protein from pigs or chickens and 10 hectares if from cattle [3,7,13].

Insects are already consumed in many parts of the world; however, there are still many obstacles regarding entomophagy in Western countries. Many people view the consumption of insects with disgust and associate it with primitive behavior. Eating behaviors and food choices are influenced by many factors such as country, living environment, sex, biology, physiology, society and culture. A large number of surveys conducted in European countries have shown that the propensity to consume EI is generally low. Even though there is a greater willingness to consume products in which insects are incorporated rather than the whole insect, there are many factors and contexts that influence the consumption of insects [3,10,11,14,15,16].

Regarding the determinants influencing the consumption of insects, some studies [10,17,18,19,20,21,22,23,24] have associated certain factors such as age, gender, country of origin, literacy about entomophagy, methods of production of products containing insects, neophobia, feeling of disgust, desire for variety and previous experiences, among others, to the willingness or refusal to eat EI.

Some research papers compiled studies focusing on the nutritional composition [7,8,25,26,27,28], health effects [12,28,29,30,31], safety [27,32], environmental sustainability [33], farming and production [27,34] of EI; however, very few have aimed to investigate the drivers that motivate the willingness to eat insects. Dagevos [35] has brought together a total of 33 studies regarding consumer research, but this review only has a small part dedicated to the benefits and barriers of eating insects, and only focusing on Western countries. In a systematic review conducted by Hartmann et al. [36], Western consumers’ perceptions and behavior regarding meat substitutes are studied. This review is mainly centered on meat substitutes as a whole, including insects and cultured meat, and only nine articles were analyzed concerning the acceptance of insects. One other review [37] has investigated pre-defined drivers of insect consumption such as ecology, subsistence strategies and social norms. 

This systematic review will bring innovation from the pre-existing reviews on this topic by providing a clear overview of the current literature regarding consumers’ perceptions, motivations, attitudes and degree of acceptance towards edible insects, not only in Western countries, but all around the world. This work aims to: (1) examine the determinants positively and negatively influencing the consumption of insects; (2) see if the degree of acceptability differs between eating whole insects and insects incorporated in products; and finally, (3) if the determinants that influence the consumption diverge between insect-eating countries (IEC) and Western countries (WC). 

## 2. Materials and Methods

### 2.1. Study Design

This systematic review was designed following the Preferred Reporting of Items for Systematic Reviews and Meta-analysis [38] and using PRISMA directives. The PRISMA 2020 Statement, published in 2021, was followed to conduct the systematic review because of its recognition as a major directive leading to high-quality reviews and to its wide usage in various areas of science [39,40].

### 2.2. Literature Search

The search was conducted in November 2021 within three databases: Web of Science, PubMed and Scopus. 

The search terms were obtained after performing a brief literature analysis. The final research expression used was ((“Edible insect*” OR Entomophagy) AND (Determinant* OR Factor* OR Predictor* OR Motiv*) AND (Consum*) AND (Attitude* OR Behavio?r* OR Accept* OR Perception*)). By conducting a search with terms such as, for example, Consum*, the databases gave back all items which contain words with the same initial letters, i.e., which are related, for example, Consumer, Consumption, Consuming, etc. On the other hand, by using search items such as Behvio?r, items with both variations of the word are picked: British (Behaviour, with u) and American (Behavior, without u). 

### 2.3. Eligibility Criteria

In this systematic review, only publications written in English and published during or after 2010 were considered. Moreover, only original articles were included, excluding book chapters, conference papers, editorial material, review articles and meta-analyses. The inclusion and exclusion criteria are summarized in Table 1.

### 2.4. Data Collection and Extraction

**Figure 1:**
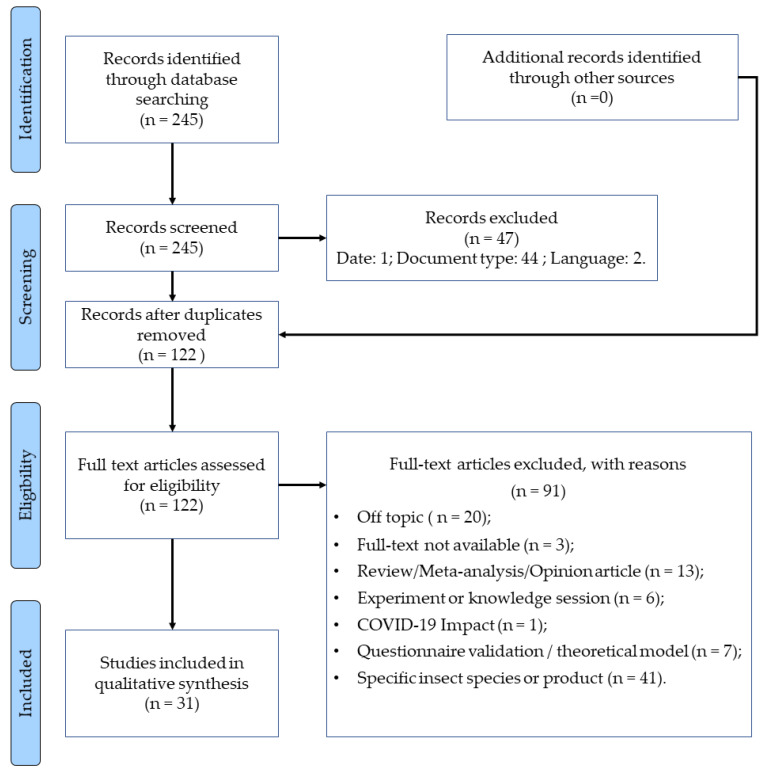
PRISMA flow diagram for the present review.

The reviewers extracted the articles into a reference manager from the databases that satisfied the eligibility criteria pre-established. The title and abstract of the articles were then read, selecting the studies that would be included in this systematic review. The other member of the research team confirmed this process. The reviewers screened the full text and either extracted the data into a standardized data collection sheet that was created beforehand, which was checked afterwards by other reviewers, or eliminated the articles that did not meet the inclusion criteria. A third reviewer was called when there was a disagreement in the study selection process. The reasons for studies’ exclusion are reported in the PRISMA flow diagram presented in Figure 1. There was no conflict of interests, for the data was based on published studies, and all the information necessary was present in the articles. 

There were two hundred and forty-five records identified through a database search and then screened, from which forty-seven records were excluded for not meeting the eligibility criteria. Duplicate entries were identified using a reference management software followed by a manual search, resulting in one hundred and twenty-two records eligible for a full-text read. From these, thirty-one were included in the study and ninety-one were excluded for the following reasons: out of scope, full text not available, not original research article, addressed the impact of COVID-19, validation problems or focusing on a very specific insect species or food containing EI rather than EI in general.

To finalize, Table A1 (presented in Appendix A) was created to summarize all the findings from the eligible studies and to gather the information that would help answer the aims of this review. 

### 2.5. Data Analysis

Some basis statistics and graphs were produced using Excel software (Microsoft Office Package, Microsoft Corporation, Redmond, Washington, EUA). 

The software VOSviewer Version 1.6.18 was used to analyze the bibliography, more specifically, to identify the co-occurrences between authors or between keywords. This software is free and can be found online at: https://www.vosviewer.com/ (accessed on 10 August 2022). This software allows for constructing and visualizing bibliometric networks. As an input, the list of references is imported to the software and from the metadata of the sources, the software produces the visualization networks. In this case, the bibliographic sources were analyzed in terms of co-occurrence and links between the keywords and also between the authors, corresponding respectively to co-citation and co-authorship relations. 

Word clouds were used to evidence the determinants of consumption of edible insects. They were obtained with WordCloud Generator—MonkeyLearn (available online at https://monkeylearn.com/word-cloud/ (accessed on 15 August 2022)). The word cloud (or tag cloud) generator allows a visual representation of words, and highlights popular words and phrases based on frequency and relevance. The relative sizes of the words relate directly to the number of occurrences of each of them.

## 3. Results

### 3.1. Bibliometric Analysis

**Figure 2:**
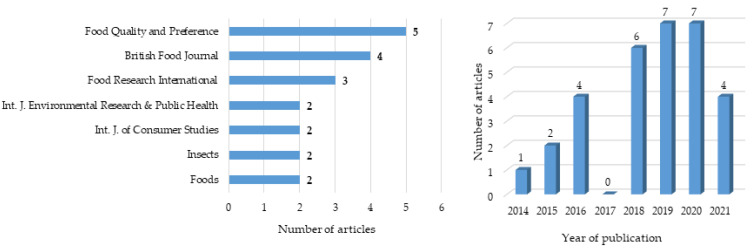
Number of articles by journal title (for journals with more than one article) and according to publication year (all 31 references).

Of the thirty-one studies selected and summarized in Table A1, six were qualitative studies and twenty-five were cross-sectional studies, with a total sample of 19,833 participants. Although the eligibility criteria in terms of publication year were to include articles from 2010 onwards, the final selected articles, following the PRISMA flow methodology, only included articles starting in 2014, as depicted in Figure 2. These numbers highlight an increase in the numbers of studies from the past to more recent years. There were only four studies reported in 2021, as the data collection was made before the end of the year, so more publications may still have been released in 2021. Figure 2 also shows the most frequent journals where the articles included in the review were published, highlighting that there were five documents from *Food Quality and Preference*, four from *British Food Journal*, three from *Food Research International*, and two from each of the following journals: *International Journal of Environmental Research and Public Health*, *International Journal of Consumer Studies*, *Insects* and *Foods*. There were eleven articles from journals that were listed only once.

**Figure 3:**
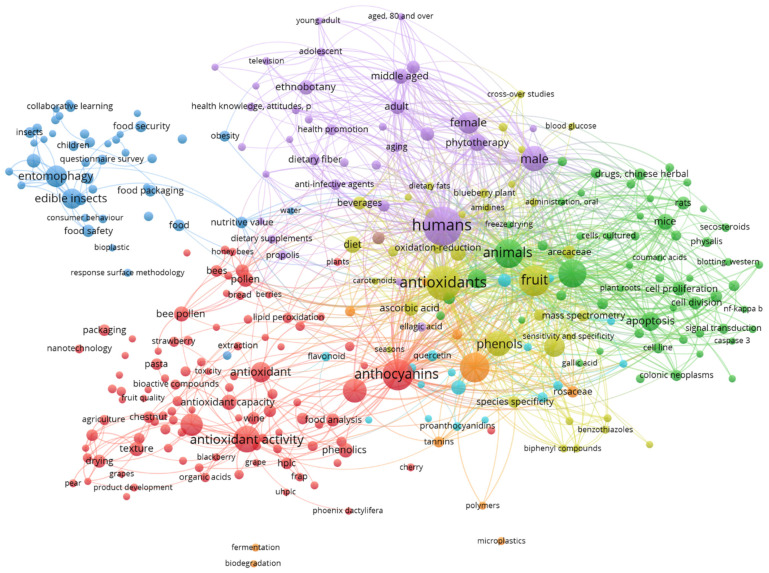
Analysis of co-occurrence links between keywords, considering those that occurred at least four times.

The thirty-one bibliographic sources included in the systematic review were analyzed using the software VOSviewer, resulting in the diagram presented in Figure 3. The diagram evidences the co-occurrence links between keywords in the bibliographic sources that occurred at least four times. In Figure 3, the size of the circles and the corresponding label represent the relative frequency of occurrence for each keyword. On the other hand, the number of sources in which the keywords occur jointly corresponds to their relatedness, which is represented by the proximity of the circles. From Figure 3, it is evident which keywords were most frequent: humans, antioxidants and flavonoids, which are linked to other keywords in the central area of the diagram that relate to the benefits for human health. Additionally, the keywords entomophagy and edible insects appear linked with questionnaire survey and consumer behavior (on the top-left side of the diagram). Finally, on the top part of the diagram, keywords including male, female, middle-aged and adult evidence some sociodemographic factors that influence the consumption of edible insects. 

**Figure 4:**
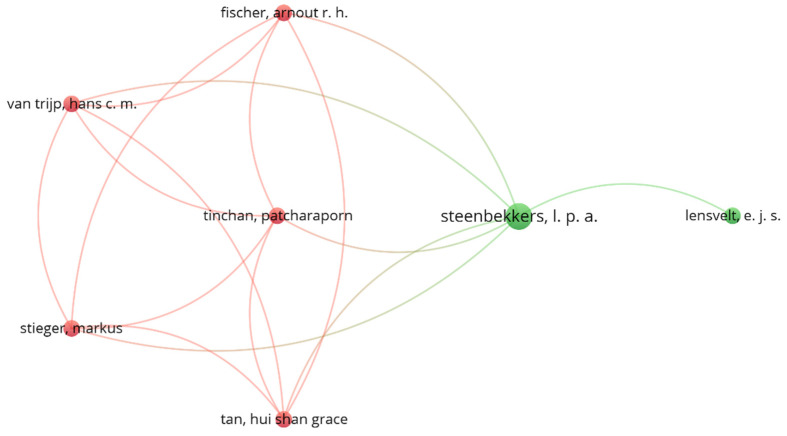
Analysis of co-authorship links between authors, considering those that occurred at least two times.

Similarly, Figure 4 presents the co-authorship links between authors in the bibliographic sources that occurred at least two times. The analysis revealed only 7 authors with links to other authors in the sources, forming 2 clusters with 16 links. Cluster One includes five authors (A. R. H. Fisher, M. Stieger, H. S. G. Tan, P. Tinchan and H. C. M. Van Trijp) and Cluster Two includes two authors (E. J. S. Lensvelt and L. P. A. Steenbekkers). These links highlight teams who work repeatedly on the topic of edible insects’ acceptance and who are major references in this field of research. Hence, they may represent a valuable source of information for this particular field of consumer science.

### 3.2. Research Characteristics

From the studies included in the review, twenty-five were cross-sectional studies [17,18,19,20,21,22,23,24,41,42,43,44,45,46,47,48,49,50,51,52,53,54,55,56]. All cross-sectional studies were supported on questionnaire surveys, while the qualitative studies were based on focus groups (five studies) [57,58,59,60,61] and interviews (one study) [62] as methodologies for data collection (Table A1). 

Regarding the sample, the studies included a highly variable number of participants, smaller in the qualitative studies, from a minimum of 13 [59] to a maximum of 54 [61] for focus groups and 77 [62] in the study by interview. In the cross-sectional studies, the sample size was also highly variable from a minimum of 88 [24] to a maximum of 7800 participants [42]. With respect to gender representativeness, the percentage of male participants varied from 13% [62] to 55% [63]. However, most studies had a smaller participation of men compared to women, and only in three studies were men the majority: 55% in Wilkinson et al. [63], 54% in Ruby et al. [23] and 53% in Lorini et al. [19] (Table A1).

With respect to the age of the participants, there was one study conducted specifically with children (4–5 years) [59] and one with seniors over sixty years [62]. Some studies were specifically for young adults: 18–24 years [47], 18–35 years [56] and 20–35 years [57]. In other studies, the participants were from all age levels, some including adolescents [20,21,43,52,53] and others including only adults starting from 18 [17,19,22,24,42,47,51,54,56,58,63], 19 [41] or 20 [57,61] years old. Some of the studies included participants, when specified, up to an age limit as high as 89 years [52,53] (Table A1). 

**Figure 5:**
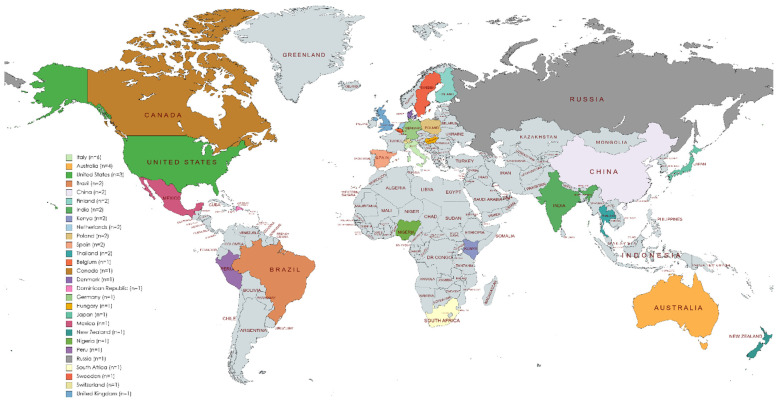
Geographical distribution and frequency of the studies included in the review.

Regarding the geographical distribution of the studies, as shown in Figure 5, most of them were conducted in European countries (Belgium, Denmark, Finland, Germany, Hungary, Italy, Netherlands, Poland, Russia, Spain, Sweden, Switzerland and United Kingdom). Some studies were also conducted in American countries (Brazil, Canada, Dominican Republic, Mexico, Peru and United States of America), African countries (Kenya, Nigeria and South Africa), Asian countries (China, India, Japan and Thailand) and Oceanian countries (Australia and New Zealand).

### 3.3. Determinants of Consumption

**Figure 6:**
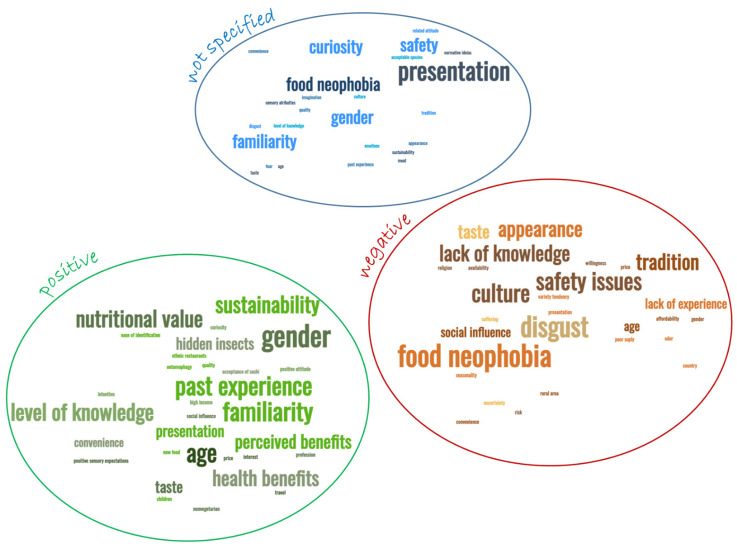
Word clouds for the determinants of consumption of EI, according to their valence: positive, negative or not specified.

This systematic review has found that there are numerous determinants influencing the consumption of EI (Table A1). They can be divided into three groups: factors that positively influence the consumption of insects, factors that negatively influence the consumption of EI and factors for which it is not specified if they have a positive or negative influence, only that they do influence the consumption of EI. Figure 6 shows the word clouds for the three groups of factors that motivate consumers towards consumption of EI. The most frequently cited positive motivations, as seen by the relative size of the corresponding words, include gender (*n* = 12, *p* = 14%), age (*n* = 8, *p* = 9%), familiarity (*n* = 8, *p* = 9%), past experience (*n* = 8, *p* = 9%), knowledge (*n* = 6, *p* = 7%), nutritional value (*n* = 6, *p* = 7%) and sustainability aspects (*n* = 6, *p* = 7%). Other motivations were also cited, but with lower numbers of occurrences and percentages. As for the most referred negative aspects, food neophobia (*n* = 8, *p* = 12%) and disgust (*n* = 7, *p* = 11%) are strongest, but safety (*n* = 5, *p* = 8%), cultural aspects (*n* = 5, *p* = 8%), tradition (*n* = 4, *p* = 6%), appearance (*n* = 4, *p* = 6%) and lack of knowledge (*n* = 4, *p* = 6%) are also relevant. Regarding the factors that were cited without specifying in which way they influence consumption of EI, the form of presentation is undoubtedly the most relevant of them, representing 10% of the reasons pointed out in the studies. 

**Figure 7:**
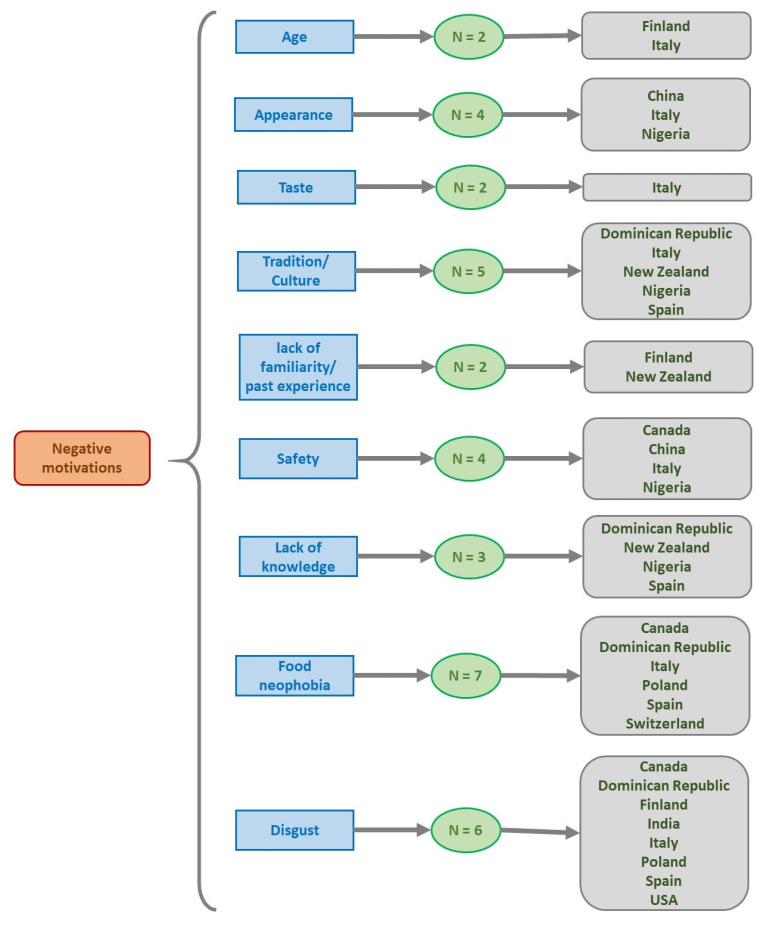
Resume of studies which report negative motivations for consumption of insects and the countries involved in the studies (N = number of studies; only if the number was two or more).

Specifically, addressing the main determinants that negatively affect the consumption of EI (Table A1), it was observed that they are related with some sociodemographic characteristics such as age [24,53], gender (females) [53], living environment (rural areas) [53] and occupation (students) [53] as well as with sensory attributes such as appearance [18,43,46,57], odor [57], taste [43,57] and presentation mode (whole insects) [45]. Some other factors that also negatively influence EI consumption are related to tradition/culture [19,44,46,57,58], social influence [57], country of origin [42], lack of familiarity/past experience [53,58], religion [23], safety [18,19,41,46], risks [23], poor supply [46], seasonality [46], price [52], lack of knowledge [44,46,58], animal suffering [23], food neophobia [17,20,24,41,44,47,50], disgust [19,20,23,41,44,52], feeling of “dirty” [58], variety-seeking tendency [20], food technology neophobia [24], intention to try [44] and finally, uncertainty [52]. Figure 7 illustrates the main negative motivations for the consumption of insects. 

**Figure 8:**
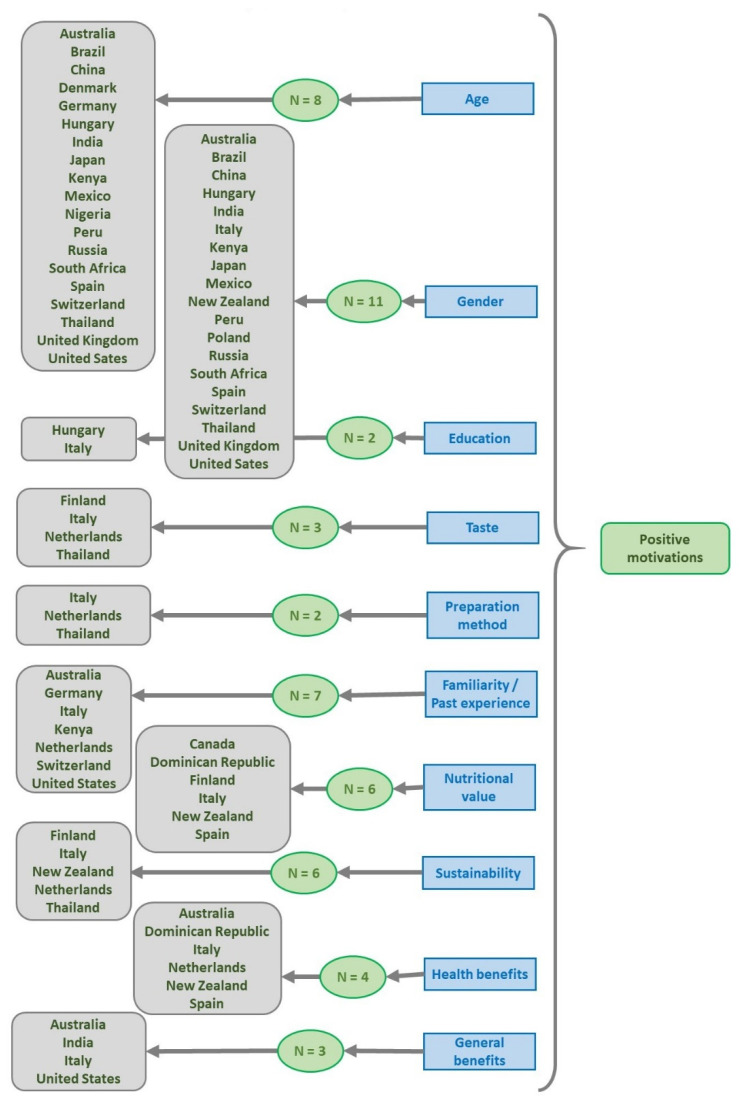
Resume of studies which report positive motivations for consumption of insects and the countries involved in the studies (N = number of studies; only if the number was two or more).

Concerning the drivers that positively correlate to the consumption of EI, these are related to age [18,21,42,46,48,50,51,55], gender (males) [17,20,23,42,43,47,48,50,51,55,58], level of education (high) [43,51], main occupation [46], level of income (high) [47], level of knowledge [17,18,19], interest in entomophagy [55], children preferences [18], hidden in food [58], quality [45], price [45], convenience [24,45], ease of identification [19], taste [22,52,61], preparation method [24,61], positive sensory expectations [50], positive attitudes to new food experiences [22,61], curiosity [52], social influence [61], intention to try [50], acceptance of sushi [23], eat in ethnic restaurants [43], familiarity/past experience [21,22,24,45,48,50,56], place of travel [47], nutritional value [19,41,44,52,57,58], sustainability [19,22,52,57,58,61], health benefits [22,44,45,58], perceived positive attributes [18] and general benefits [23,24,63]. Figure 8 gives a brief description of these factors that positively influence the consumption of EI.

As previously mentioned, some studies also discuss which determinants affect the consumption of insects; however, they do not differentiate between positive and negative drivers. The factors that are mentioned in these studies are related with age [54], gender [49,54], social influence [60], familiarity [54,63], level of knowledge [62], tradition/culture [62], disgust [62], curiosity [59,62], fear [59], normative ideas [59], food neophobia [54,63], emotions [59], imagination [59], sensory attributes [62], safety [60,62,63], acceptable species [62], presentation [49,62,63], taste [60,63], availability [60], convenience [54,60], affordability [60], benefits beyond nutritional [60], sustainability [54], meat-related attitudes [54], appearance [63] and quality [63].

### 3.4. Whole Insects (WI) versus Food Containing Insects (FCI)

**Figure 9:**
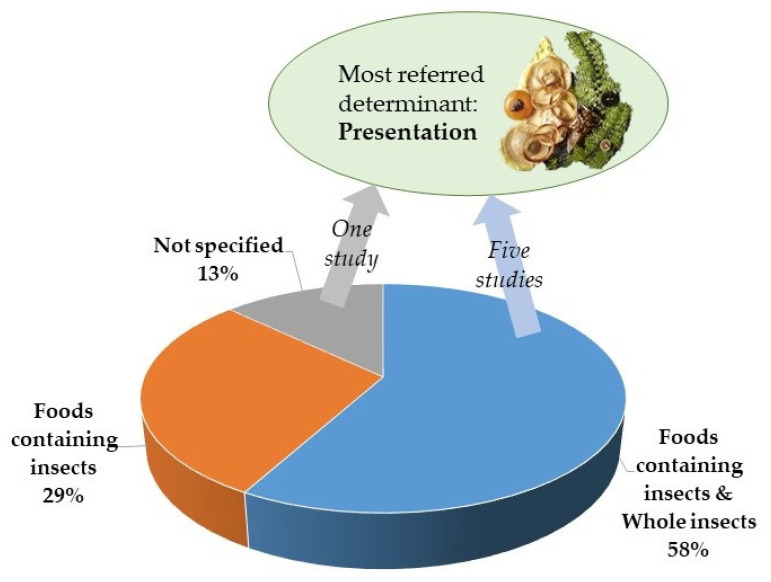
Distribution of studies according to the form of the insects: disguised in foods or whole.

In this systematic review, eighteen studies focused on the consumption of both WI and FCI [17,18,19,20,21,23,41,43,45,49,50,52,55,57,58,59,61,63], nine focused only on FCI [22,24,42,48,51,53,54,56,60] and four did not specify whether they referred to the consumption of WI or FCI [44,46,47,62] (Figure 9).

Of the studies that focused on the consumption of insects as a whole plus their incorporation into foods, five studies [45,49,58,61,63] have shown that the way insects are presented to consumers is an important determinant of consumption. Moreover, these have highlighted that people have a more positive perception towards edible insects when they are masked or hidden in foods rather than when they are consumed whole.

One other study [62], in which it was not specified if the acceptance of insects was towards whole insects or insect-based food, has shown that presentation is one of the factors that condition the willingness to consume EI. 

Overall, from all the studies considered, it was concluded that, regarding the preference towards foods that contain insects as ingredients or whole insects, was related primarily to the presentation, which is, therefore, a major determinant for acceptance, as evidenced in six of the studies (Figure 9).

### 3.5. Insect-Eating Countries (IEC) versus Western Countries (WC)

This paper brings together scientific research that is collected in insect-eating countries, in Western countries or, in some cases, in countries within which insects are part of the food culture and tradition in some places but not in others. The studies included in this systematic review were mostly carried out on Western countries (68%), with a minor representation of studies on insect-eating countries (6%). However, there were also a significant number of studies that were conducted in both (26% of the studies).

Concerning the determinants of consumption of EI, it was observed that there are some factors that are exclusive for IEC. The positive drivers found are related to the perceived positive attributes [18], children’s preferences [18] and main occupation [46], whereas the negative factors are related to poor supply [46] and seasonality [46]. 

On the other hand, some determinants are particular to WC, specifically those related to nutritional value [19,41,44,52,57,58], eating in ethnic restaurants [43], insects being hidden in food [58], price [45], ease of identification [19], income level [47], place of travel [47], acceptance of sushi [23], intention to try [50], positive sensory expectations [50], interest in entomophagy [55], odor [57], social influence [57], food neophobia [17,20,24,41,44,47,50], disgust [19,20,23,41,44,52], feeling of “dirty” [58], variety-seeking tendency [20], risks [23], suffering [23], religion [23], food technology neophobia [24], uncertainty [52], fear [59], living environment [53], normative ideas [59], imagination [59], emotions [59] and meat-related attitudes [54].

Furthermore, there are some positive determinants that are common to both WC and IEC, such as age [18,21,42,46,48,50,51,55], gender (males) [17,20,23,42,43,47,48,50,51,55,58], level of education [43,48,51], main occupation [46], level of knowledge [17,18,19], taste [22,52,61], preparation method [24,61], positive attitudes to new food experiences [22,61], social influence [61], familiarity/past experience [21,22,24,45,48,50,56], sustainability [19,22,52,57,58,61], health benefits [22,44,45,58], perceived positive attributes [18] and general benefits [23,24,63]. As for the negative factors that are in common to both WC and EIC, they are related with appearance [18,43,46,57], tradition/culture [19,44,46,57,58], country of origin [42], safety [18,19,41,46] and lack of knowledge [44,46,58]. Regarding the factors that are not specified to be positive nor negative, these are related with gender [49,54], social influence [60], familiarity [54,63], level of knowledge [62], tradition/culture [62], disgust [62], curiosity [59,62], food neophobia [54,63], sensory attributes [62], safety [60,62,63], acceptable species [62], presentation [49,62,63], taste [60,63], availability [60], convenience [54,60], affordability [60], benefits beyond nutritional [60], appearance [63] and quality [63]. 

**Figure 10:**
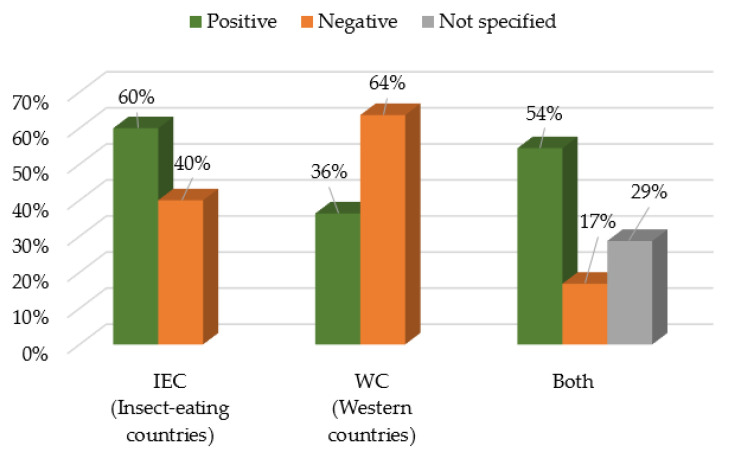
Motivations for the consumption of insects according to being traditional or not traditional to eat insects.

Figure 10 shows the relative proportion of the positive and negative factors that influence consumption depending on the countries’ traditions. It is observed that in insect-eating countries, there are more positive determinants of consumption than negative (60% against 40%). On the other hand, in the Western countries, an opposite trend is verified, with only 36% of the determinants being favorable to the consumption while 64% are against it. In the case of studies which include both categories of countries, there is also a predominance of the positive determinants for consumption (54%), although there is also an expressive percentage of determinants for which it was not specified in the studies whether they are in favor of or contrary to the consumption of EI (29%). 

## 4. Discussion

In the present systematic review, thirty-one studies were gathered in order to assemble the main determinants that influence the consumption of edible insects. This study has also highlighted the differences that exist between insect-eating counties and Western countries, as well as the degree of acceptability between whole insects and food containing insects. The review by Mishyna et al. [64] showed that the sensory characteristics of edible insects are major drivers for consumer appeal. The improvement of the image of EI is one valuable strategy to increase their acceptability, regardless of whether or not their consumption is a common practice among a certain population [65].

A total of 57 different factors influencing the consumption of EI were observed (acceptable species; acceptance of sushi; affordability; age; appearance; availability; benefits; children’s preferences; convenience; country of origin; curiosity; disgust; ease of identification; eat in ethnic restaurants; emotions; familiarity/past experiences; fear; feeling of “dirty”; food neophobia; food technology neophobia; gender; health benefits; hidden in food; income level; imagination; intention to try; interest in entomophagy; level of education; level of knowledge; living environment; main occupation; meat-related attitudes; normative ideas; nutritional value; odor; perceived positive attributes; place of travel; poor supply; positive attitudes to new food experiences; positive sensory expectations; preparation method; presentation; price; quality; religion; risks; safety; seasonality; sensory attributes; social influence; students; suffering; sustainability; taste; tradition/culture; uncertainty and variety-seeking tendency). Of all these determinants, the positive factors that are mentioned most are age, gender, nutritional value, sustainability and familiarity/past experience. On the other hand, the negative factors that are mentioned most are food neophobia, disgust, tradition/culture and appearance. These findings are in agreement with a literature review [35] that demonstrates that sensory appeal (taste), price, food literacy, appropriateness, food culture, nutritional value, food safety, ethical and health reasons and sustainability concerns are important determinants in consumers’ willingness to eat insects in Western countries. Sustainability is one of the factors that can be used to help motivate new consumers into trying insect-based foods. Because it has been shown that insects are less demanding in terms of production factors, require the use of less resources and contribute to biodiversity [21,66,67,68], they are possible replacements for those consumers that tend to adopt diets that avoid traditional meats, such as, for example, vegetarians.

The results have shown that age is mentioned in eleven of the thirty-one studies; however, there is some divergence regarding the age group that is most to consume insects. Eight of the studies [18,21,42,46,48,50,51,55] demonstrated that age is a positive determinant influencing the consumption of EI. Of the eight studies, one [42] indicates that the most age group is 18 to 54 years old, two [18,46] do not suggest a specific age group, whereas four [21,48,50,55] indicate that the youngest are the most willing consumers of insects. Moreover, two studies [24,53] have shown age as being a negative factor influencing the consumption of insects. Interestingly, one of these studies [24] shows that the eldest are the age group least willing to consume insects, which is concordant with the previous studies. However, one of the studies [53] differs from the others by stating that those under 25 years old are the age group least eager to eat insects. Furthermore, there is one study [54] out of the eleven that demonstrates that age is a determinant of consumption; however, it does not indicate if it is a positive or negative determinant and, thus, there is no evidence of which age group is most or least willing to consume EI.

It was observed in this systematic review that a total of fifteen studies [17,20,23,42,43,47,48,49,50,51,52,53,54,55,58] have demonstrated gender to be a factor influencing the consumption of EI. Of these, twelve [17,20,23,42,43,47,48,50,51,52,55,58] have shown that males are more willing to try and consume EI than females. However, from those, only one study [53] indicates gender as a negative factor. Of the fifteen, two studies [49,54] say that gender is a determinant but do not mention which gender is more willing to eat insects. However, there is one study [42], in which the data were collected in different countries, that shows that one country, China, does not follow this tendency. In this study, it was found that in China, females are more willing to eat insect than males, contrary to all other countries. These findings are not in line with other studies such as, for example, the study by Hartmann et al. [69] where Chinese women were found to be less likely to accept insects as food than Chinese men. In a recent cross-cultural study, promoted between WC, it was shown that gender had a significant impact on entomophagy acceptance for Portuguese consumers, while it had no such impact for Norwegian consumers [70], the former probably related to males having lower disgust sensitivity than women [23].

Pertaining to the education level, three studies [43,48,51] have shown that education is one of the determinants influencing the consumption of edible insects. Two of the studies [43,51] verified that people with higher education level are more willing to consume insects; however, the third study [48] states the opposite, highlighting that people with a lower level of education are more familiar with the practice of entomophagy. This finding may be related to the first two studies being in countries (Italy and Hungary) where the consumption of insects is not a common practice, whereas in the third study (Kenya), entomophagy is a common practice in the household, and so people with lower education levels tend to continue following the gastronomic traditions of the country. According to Liceaga et al. [71], the level of neophobic response to EI is variable amongst consumers depending on a number of sociodemographic and cultural factors, including education or social status.

Concerning the determinant taste, this systematic review has shown that three studies [22,52,61] consider this a positive driver, whereas two studies [43,57] have indicated this to be a negative factor influencing the consumption of edible insects. The sensory properties of food are very important for acceptance; hence, the taste is a crucial motivator of consumer appeal to eating insects [36]. Mishyna et al. [64] discuss the sensory and visual properties of EI and FCI as a way to enhance consumer appeal. A work on the sensory profiling of insect-containing food products, performed by Ribeiro et al. [72], has shown that a simple process such as defatting freeze-dried edible crickets (*Acheta domesticus* and *Gryllodes sigillatus*) and their incorporation into a snack bar would have a dramatic impact on the sensory profiling, with a significant positive increase in both liking and acceptance. The improvement of the sensory characteristics of insect foods includes manipulation of flavor and texture, while appearance must also be worked on due to its pivotal role. Culinary and technological operations also influence the characteristics and consumer acceptability. Finally, promoting familiarity will also improve willingness to consume EI.

One of the most prominent factors affecting the willingness of consumers towards EI is food neophobia. Food neophobia is the propensity that people have to avoid unfamiliar foods and/or have an aversion towards new foods. A high level of food neophobia is often associated with lower willingness to try new foods. For example, in countries where EI do not belong in the traditional diet, there is a reluctance to eat unfamiliar foods, many times due to the negative associations that are made [35,73,74,75,76]. The present review has shown that consumers are more eager to try FCI rather than the WI. This idea is supported by Lammers et al. [77], who indicate that consumers are more readily willing to adopt eating invisible insects in familiar-looking and familiar-tasting foods than accepting the whole insects. However, Tan et al. [78] refer that, although incorporating novel food ingredients such as EI into familiar products could help to create more positive expectations, these are still less appealing than the original products that consumers are used to.

Food habits and food choices are different all around the world; however, globalization has brought together many cultures and gastronomic traditions. As already mentioned, insects are eaten in many parts of the world. For example, in Thailand and in China, insects are commonly consumed and found in restaurant menus. In opposition, in WC, people tend to show phobia and disgust towards the consumption of insects [73]. The work by Guiné et al. [79] discusses the path from insects as ethnic food into novel foods. It is referred that insects constitute a basic food for many communities, providing livelihood and also making part of the social context by being consumed in festivals and religious occasions, for example.

This systematic review has also aimed to understand the differences between WC and IEC, having found that there are some determinants that are exclusive to IEC, such as poor supply, seasonality, children’s preferences in the household, perceived positive attributes and main occupation. Some factors are also limited to WC, more specifically, these are related to nutritional value, eat in ethnic restaurants, hidden in food, price, ease of identification, income level, place of travel, acceptance of sushi, intention to try, positive sensory expectations, interest in entomophagy, odor, social influence, food neophobia, disgust, feeling of “dirty”, variety-seeking tendency, risks, suffering, religion, food technology neophobia, uncertainty, fear, living environment, normative ideas, imagination, emotions and meat-related attitudes. Our findings show that, whereas in IEC, there is a greater focus on factors related to positive sensory attributes, availability, affordability and preferences, in WC, there is a bigger emphasis on determinants such as nutritional value, sustainability, benefits, familiarity/past experience, tradition/culture, food neophobia and disgust. These findings are corroborated by many studies focusing on the acceptance of EI and foods containing insects in diverse Western countries [75,80,81]. 

The differences between IEC and WC regarding the willingness to accept EI and the determinants of consumption are associated with lifestyles, societal variables, culture and tradition. A study by Sato and Ishizuka [82] investigated the influence of traditional insect food culture on the acceptance of novel insect foods in two populations with different backgrounds on entomophagy. They found that, despite the role of tradition as a motivator for the consumption of insects (also in cultures where eating insects is not usual), the willingness to have them arises due to different reasons such as, for example, the sustainability of their diets. On the other hand, a number of studies point out that acceptance of food products with EI can be more difficult in countries where they are not traditionally consumed [83,84].

Nevertheless, as stated by Ribeiro et al. [70], the social and cultural norms surrounding edible insects need to change in order for them to be successfully implemented in Western food market.

## 5. Conclusions

Entomophagy, even though considered a common practice in many countries, still presents a major challenge for Western countries. Thus, there is a need to normalize the consumption of insects in countries where this tradition is not part of the food culture. To our knowledge, this was the first study that gathered the current literature regarding consumers’ motivations, attitudes and perceptions towards EI, investigating what the main determinants of consumption are, and if there are differences between WC and IEC and between WI and FCI. 

This work has highlighted the main determinants positively and negatively influencing the consumption of EI. It was found that people with a higher willingness to consume edible insects are young males with a high level of education and high level of knowledge towards EI, with some degree of curiosity, intention to try, familiarity or past experience, that eat in ethnic restaurants and enjoy sushi, with low food neophobia and disgust, with positive attitudes to new foods, an interest in entomophagy and that care about issues such as nutritional value, sustainability, health benefits, sensory attributes, social influence and presentation.

Moreover, it was observed that the main factors influencing insect acceptability differ between WC and IEC. While, in IEC, there is a greater focus on factors related to sensory attributes, availability and affordability, in WC, the emphasis is on determinants such as nutritional value, sustainability, benefits, familiarity/past experience, tradition/culture, food neophobia and disgust.

Finally, it was concluded that people are more willing to accept food products containing insects rather than the whole insect, which is one of the most promising points to be addressed in the future. 

Hence, some key finding of this work can contribute to enhancing the existing knowledge on determinants of acceptance of EI as food by populations with different cultural backgrounds. More specifically, the acceptance of EI in countries without tradition of entomophagy is more difficult, but depends on some positive motivators, including concerns about sustainability, desire to try new foods and curiosity. Still, it is evidenced that acceptance can be greatly increased regarding foods that contain insects as hidden ingredients, as opposed to eating the whole insects. This information can be valuable for the intervening bodies to design strategies to incentivize the adoption of EI. 

Based on these findings, the upcoming research should focus on marketing strategies and community-based interventions aiming to inform consumers, modify behaviors, transform normative ideas and create appealing new food products utilizing edible insects.

One other relevant aspect that could be improved in the future is to increase the number of studies which include countries where eating insects is a culturally accepted practice, since they are presently scarce. Furthermore, studies in which direct comparisons are made between different countries are lacking in the scientific literature; therefore, more studies of this nature should be conducted.

Although bringing valuable systematized information to the scientific literature, this review has some limitations that are worth mentioning. Many studies had to be excluded due to not meeting the inclusion criteria predefined, and so the findings do not assemble all the information regarding the motivations, attitudes, perceptions and determinants of the consumption of edible insects. Furthermore, this review did not analyze the risk of bias, thus possibly influencing the results presented.

## Figures and Tables

**Table 1:** Inclusion and exclusion criteria.

**Inclusion Criteria**
English; Published during or after 2010; Original articles; Focus on factors, determinants and drivers of EI consumption.
**Exclusion Criteria**
Book chapters, conference papers, editorial material, review articles, meta-analyses, opinion articles; Off topic (Animal feed/no mention of EI/no consumption of EI/market perspective/ nutrition/health/sensory analysis, etc.); Full text not available; Experiments/knowledge sessions; COVID-19 impact; Questionnaire validation/theoretical models; Focus on a specific insect species or food containing EI.

## Data Availability

Data are available from the corresponding author upon reasonable request.

## Appendix A

**Table A1:** Summary of the studies included in the review.

(Author, Year)	Study Design ^a^	Country (Region)	Insect-Eating Country? ^b^	Instrument for Data Collection	Sample Size and Composition ^c^	Form of the Insect ^d^	Factors Influencing Insect Acceptance
(Balzan et al., 2016) [57]	QS	Italy	No	Focus group	32 participants (11 M, 21 F) (20 to 35 y)	W + F	**Positive** Nutritional value; Sustainability (low space requirement and ecologically compatible). **Negative** Appearance; Odor; Taste; Tradition/Culture; Social influence.
(Barton et al., 2020) [41]	CSS	Canada (Nova Scotia)	No	Questionnaire	100 participants (39 M, 61 F) (19 to 69 y)	W + F	**Positive** Nutritional value. **Negative** Food neophobia; Disgust; Safety (source of toxins, infectious diseases and harmful microbes).
(Castro et al., 2019) [42]	CSS	United Sates, Mexico, Peru, Brazil, United Kingdom, Spain, Russia, India, China, Thailand, Japan, South Africa and Australia	B	Questionnaire	7800 participants (630 recruited per country) (18 to 55+ y)	FCI	**Positive** Gender (males more willing than females in all countries except China); Age (18 to 54 more willing than 55+). **Negative** Country (Western countries less willing to try than other countries).
(Cicatiello et al., 2016) [43]	CSS	Italy	No	Questionnaire	201 participants (90 M, 111 F) (14 to 78 Y)	W + F	**Positive** Gender (males more willing than females); High education; Eat in ethnic restaurants. **Negative** Appearance; Taste.
(Clarckson et al., 2018) [58]	QS	New Zealand	No	Focus group	32 participants (9 M, 23 F) (18 to 75 y)	W + F	**Positive** Gender (males more willing than females); Nutritional value; Health benefits; Sustainability; Hidden insects in food. **Negative** Culture; Feeling of “dirty”; Lack of knowledge; Lack of past experience.
(Gómez-Luciano, 2021) [44]	CSS	Spain (S) Dominican Republic (DR)	No	Questionnaire	401 participants	NS	**Positive** Nutritional value; Health benefits. **Negative** Intention to try; Disgust; Food neophobia; Lack of knowledge; Tradition/Culture.
(Laureati et al., 2016) [17]	CSS	Italy	No	Questionnaire	341 participants (118 M, 223 F) (18 to 80 y)	W + F	**Positive** Gender (males more willing than females); Level of knowledge. **Negative** Food neophobia.
(Levesvelt et al., 2014) [45]	CSS	Australia Netherlands	No	Questionnaire	208 participants (74 Australian, 134 Dutch)	W + F	**Positive** Familiarity/Past experience; Health benefits; Quality; Price; Convenience. **Negative** Presentation (Whole insects).
(Liu et al., 2020) [18]	CSS	China	Yes	Questionnaire	614 participants	W + F	**Positive** Perceived positive attributes; Children preferences; Age; Level of knowledge. **Negative** Appearance; Safety.
(Lorini et al. 2021) [19]	CSS	Italy (Florence)	No	Questionnaire	248 participants (132 M, 116 F) (18 to 38 y)	W + F	**Positive** Nutritional value; Sustainability; Ease of identification; Level of knowledge. **Negative** Safety; Tradition/Culture; Disgust.
(Meludu et al., 2018) [46]	CSS	Nigeria (Kogi State)	Yes	Questionnaire	160 participants	NS	**Positive** Age; Main occupation. **Negative** Tradition/Culture; Poor supply; Safety (lack of hygiene); Appearance; Seasonality; Lack of knowledge.
(Modlinska et al., 2021) [20]	CSS	Poland	No	Questionnaire	1096 participants (493 M, 603 F) (16 to 78 y)	W + F	**Positive** Gender (males more willing than females). **Negative** Food neophobia; Disgust; Variety-seeking tendency.
(Myers et al., 2018) [62]	QS	Australia	B	Interview	77 participants (10 M, 67 F) (60+ y)	NS	**General (not specified)** Disgust; Tradition/Culture; Sensory attributes; Safety; Acceptable species; Presentation (Whole or in food); Curiosity; Level of knowledge.
(Nyberg et al. 2021) [59]	QS	Sweden	No	Focus group	13 participants (5 M, 8 F) 4 to 5 y)	W + F	**General (not specified)** Curiosity; Fear; Normative ideas; Emotions; Imagination.
(Orkusz et al., 2020) [47]	CSS	Poland (Wroclaw)	No	Questionnaire	454 participants (18 to 24 y)	NS	**Positive** Higher income level; Place of travel (North or South America and Asia); Gender (males more willing than females). **Negative** Food neophobia.
(Orsi et al., 2019) [21]	CSS	Germany	No	Questionnaire	393 participants (193 M, 200 F) (13 to 82 y)	W + F	**Positive** Age (youngest); Familiarity/>Past experience; Non-vegetarian; Health benefits. **Negative** Food neophobia; Disgust.
(Palmieri et al., 2019) [22]	CSS	Italy (Abruzzo, Campania, Lazio and Molise)	B	Questionnaire	456 participants (146 M, 310 F) (18 to 65 y)	FCI	**Positive** Positive attitude to new food experiences; Taste; Health benefits; Sustainability; Familiarity/Past experience;
(Pambo et al., 2016) [60]	QS	Kenya (Siaya, Vihiga and Machakos)	B	Focus group	43 participants (15 M, 28 F)	FCI	**General (not specified)** Taste; Availability; Convenience; Affordability; Benefits beyond nutrition; Social influence; Safety.
(Pambo et al., 2018) [48]	CSS	Kenya	B	Questionnaire	432 participants	FCI	**Positive** Age (youngest); Gender (males more willing than females); Level of education (lower); Familiarity/Past experience.
(Ruby et al., 2019) [23]	CSS	USA India	No	Questionnaire	275 participants (USA) (124 M, 151 F) 201 participants (India) (133 M, 68 F)	W + F	**Positive** Acceptance of sushi; Gender (males more willing than females); Aware of the benefits. **Negative** Risks; Disgust; Suffering; Religion.
(Schardong et al., 2019) [49]	CSS	Brazil (North, Northeast, Midwest, Southeast and South)	B	Questionnaire	1619 participants (608 M, 1011 F) (0 to 50+ y)	W + F	**General (not specified)** Gender (males more willing than females); Presentation (more willing to accept FCI).
(Schlup et al., 2018) [50]	CSS	Switzerland	No	Questionnaire	379 participants (174 M, 205 F)	W + F	**Positive** Age (youngest); Gender (males more willing than females); Familiarity/Past experience; Intention to try; Positive sensory expectations. **Negative** Food neophobia;
(Sogari et al., 2019) [24]	CSS	Italy (Parma)	No	Questionnaire	88 participants (43 M, 45 F) (18 to 40 y)	FCI	**Positive** Preparation method (more willingness to try FCI than whole insects); Familiarity/Past experience; Aware of the benefits; Convenience. **Negative** Age (oldest); Food neophobia; Food technology neophobia.
(Szendro et al., 2020) [51]	CSS	Hungary	No	Questionnaire	414 participants (143 M, 271 F) (18 to 50+ y)	FCI	**Positive** Age (30–39 y); Gender (males more willing than females); Graduates.
(Tan et al., 2015) [61]	QS	Thailand (T) Netherlands (N)	B	Focus group	54 participants (19 M, 35 F) (20 to 65 y)	W + F	**Positive** Sustainability; Taste; Familiarity/Past experience; Social influence; Preparation method (more willingness to try FCI than whole insects).
(Tuccillo et al., 2020) [52]	CSS	Finland	No	Questionnaire	567 participants (188 M, 379 F) (16 to 89 y)	W + F	**Positive** Gender (males more willing than females); Sustainability; Nutritional value; Curiosity; Taste. **Negative** Disgust; Price; Uncertainty.
(Vartiainen et al., 2020) [53]	CSS	Finland	No	Questionnaire	567 participants (188 M, 379 F) (16 to 89 y)	FCI	**Negative** Gender (females); Students; Age (under 25 y) Living environment (rural areas); No familiarity/Past-experiences.
(Verbeke, 2015) [54]	CSS	Belgium (Flanders)	No	Questionnaire	368 participants (143 M, 225 F) (18 to 79 y)	FCI	**General (not specified)** Gender; Age; Familiarity; Food neophobia; Convenience; Sustainability; Meat-related attitudes.
(Videbaek et al., 2020) [55]	CSS	Denmark	No	Questionnaire	975 participants (479 M, 496 F)	W + F	**Positive** Age (youngest); Gender (males); Interest in entomophagy.
(Wilkinson et al., 2018) [63]	CSS	Australia	B	Questionnaire	820 participants (451 M, 369 F) (18 to 65+ y)	W + F	**General (not specified)** Taste; Appearance; Safety; Quality; Food neophobia; Familiarity/Past experience; Presentation (more willingness to try FCI than whole insects).
(Woolf et al., 2019) [56]	CSS	USA	No	Questionnaire	397 participants (136 M, 261 F) (18 to 35+ y)	FCI	**Positive** Familiarity/Past experience; Aware of the benefits.

^a^ QS = Qualitative study; CSS = Cross-sectional study. ^b^ Yes = Insects are part of the local food culture; No = Insects are not part of the food culture; B = In some parts of the country, insects are part of the local food culture, and in others, they are not OR, in case of multicounty studies, B = In some countries, insects are part of the local food culture, and in others, they are not. ^c^ M = masculine, F = feminine, y = years. ^d^ WI = Whole insect; FCI = Food containing insects; W + F = Whole insects plus food containing insects; NS = Not specified.
